# Morphometric features of gastric mucosa in atrophic gastritis: A different pattern between corpus and antrum

**DOI:** 10.1097/MD.0000000000033480

**Published:** 2022-04-07

**Authors:** Xue-Mei Lin, Li Wang, Chun-Hui Xi, Jun Wang, Xian-Fei Wang, Qiong Wang, Cong Yuan

**Affiliations:** a Department of Pathology, Institute of Basic Medicine and Forensic Medicine, North Sichuan Medical College, Nanchong, China; b Department of Pathology, Affiliated Hospital of North Sichuan Medical College, Nanchong, China; c Department of Gastroenterology, Affiliated Hospital of North Sichuan Medical College, Nanchong, China.

**Keywords:** atrophic gastritis, diagnostic performance, full-thickness gastric mucosa, histopathology, morphometry

## Abstract

Atrophic gastritis can cause mucosa thinning, while detailed metrological evidence is lacking. We aimed to compare the morphological features of full-thickness gastric mucosa in antrum and corpus and evaluate the diagnostic performance for atrophy. Gastric cancer patients were prospectively enrolled (N = 401). Full-thickness gastric mucosa was obtained. Foveolar length, glandular length and musculus mucosae thickness were measured. Pathological assessment was conducted using the visual analogue scale of the updated Sydney system. Areas under the receiver operating characteristic curves (AUCs) were calculated for different atrophy degrees. In corpus mucosa, foveolar length and musculus mucosae thickness were positively correlated with the atrophy degree (spearman’s correlation coefficient [*r*_s_] = 0.231 and 0.224, respectively, *P* < .05); glandular length and total mucosal thickness were negatively correlated (*r*_s_ = −0.399 and −0.114, respectively, *P* < .05). Total mucosal thickness did not correlate with antral atrophy degree (*P* = .107). The AUCs of total mucosal thickness for corpus and antral atrophy were 0.570 (*P* < .05) and 0.592 (*P* < .05), respectively. The AUCs for corpus atrophy, moderate and severe, and severe atrophy were 0.570 (*P* < .05), 0.571 (*P* = .003), and 0.584 (*P* = .006), respectively. The corresponding AUCs for antral atrophy were 0.592 (*P* = .010), 0.548 (*P* = .140), and 0.521 (*P* = .533), respectively. The tendency for mucosal thickness to thin with atrophy occurred in the corpus rather than in the antrum. The diagnostic performance of corpus and antral mucosal thickness was limited for atrophy.

## 1. Introduction

Atrophic gastritis has represented a common condition, mainly caused by *Helicobacter pylori* infection.^[1,2]^ Atrophic gastritis is a chronic process, resulting in the “appropriate glands” to decrease or even disappear completely after years of mucosal inflammation.^[3,4]^ It is widely accepted that atrophic gastritis can cause mucosa thinning and increase the visibility of submucosal blood vessels during gastroscopy.^[1,4–7]^

The endoscopists typically discern atrophic mucosa based on the endoscopic atrophic signs, such as loss of gastric rugal folds, pale mucosal appearance, the height difference of gastric mucosa and pattern of visible vessels.^[1,5–10]^ However, several studies^[8,10–13]^ implied that endoscopic atrophic signs were poorly consistent with pathological findings. Previous studies showed that white light gastroscopy had only 42% sensitivity for atrophic gastritis,^[14]^ demonstrating that the majority of atrophic mucosa did not show signs of endoscopic atrophy. On the contrary, in the clinical practice, we noticed that although some gastric mucosa showed submucosal vascular pattern, pathological findings confirmed no mucosal atrophy. Eshmuratov et al^[15]^ also found that the accuracy of submucosal vascular pattern in the diagnosis of corpus and antral histological atrophy was 65.0% and 59.9%, respectively, suggesting that a considerable proportion of gastric mucosa thinning did not indicate the presence of atrophy. In view of the uneven distribution of atrophic mucosa,^[1,8,16]^ the endoscopic atrophic signs did not correspond precisely to the biopsy sites in the previous studies, which could lead to unreliable results. Thus, we intended to conduct a metrological evaluation in the area of interest of gastric mucosal pathological sections, and explore the association between mucosal thickness and atrophy.

Endoscopic biopsy is the principal method to obtain the gastric tissues. This method is insufficient to obtain the full-thickness mucosa due to the limitation of biopsy depth.^[8,17,18]^ At present, the metrological results of atrophic gastritis are mainly based on a small number of biopsy specimens and only confined to the foveolae and glandular layer, not including the musculus mucosae.^[18,19]^ So, the aim of this study is to obtain intact full-thickness mucosa from surgical stomach specimens to investigate the morphometric features of atrophic gastritis and evaluate the diagnostic performance for atrophy.

## 2. Materials and methods

### 2.1. Surgical gastric specimen population

The prospective study was conducted at the Affiliated Hospital of North Sichuan Medical College. We consecutively enrolled gastric cancer inpatients between August 2021 and July 2022. The patients who underwent partial or total gastrectomy were eventually included in the study. The exclusion criteria were as follows: patients with prior history of gastrectomy or endoscopic submucosal dissection, patients with linitis plastica and mucosa-associated lymphoid tissue lymphoma, and patients with a medical history of previous or current chemoradiotherapy. The protocol was approved by the Ethics Committee of North Sichuan Medical College (NSMC-2021-05). Informed consent was obtained from all patients.

### 2.2. Surgical gastric specimen collection

Each stomach was processed immediately after resection. Freshly stomachs were opened along the greater curvature when necessary, and bathed in the 10% neutral formalin solution for 24 to 48 hours at room temperature. For the stomach with distal gastrectomy, 3 full-thickness gastric wall tissues with approximately 1.5 cm long were taken at the antrum 3 to 5 cm from the pylorus. For the stomach with proximal gastrectomy, 3 tissues with similar size were obtained from the corpus. For the stomach with total gastrectomy, 3 tissues were acquired from the above-mentioned regions of the corpus and antrum, respectively. All sampling areas were located in the grossly normal gastric mucosa and more than 1 cm away from the naked-eye tumor site. Stomach specimens would be discarded if a suitable sampling site was not available. One section of each tissue was used for subsequent evaluation.

### 2.3. Determination of the region of interest and morphometric assessment

**Figure 1.**
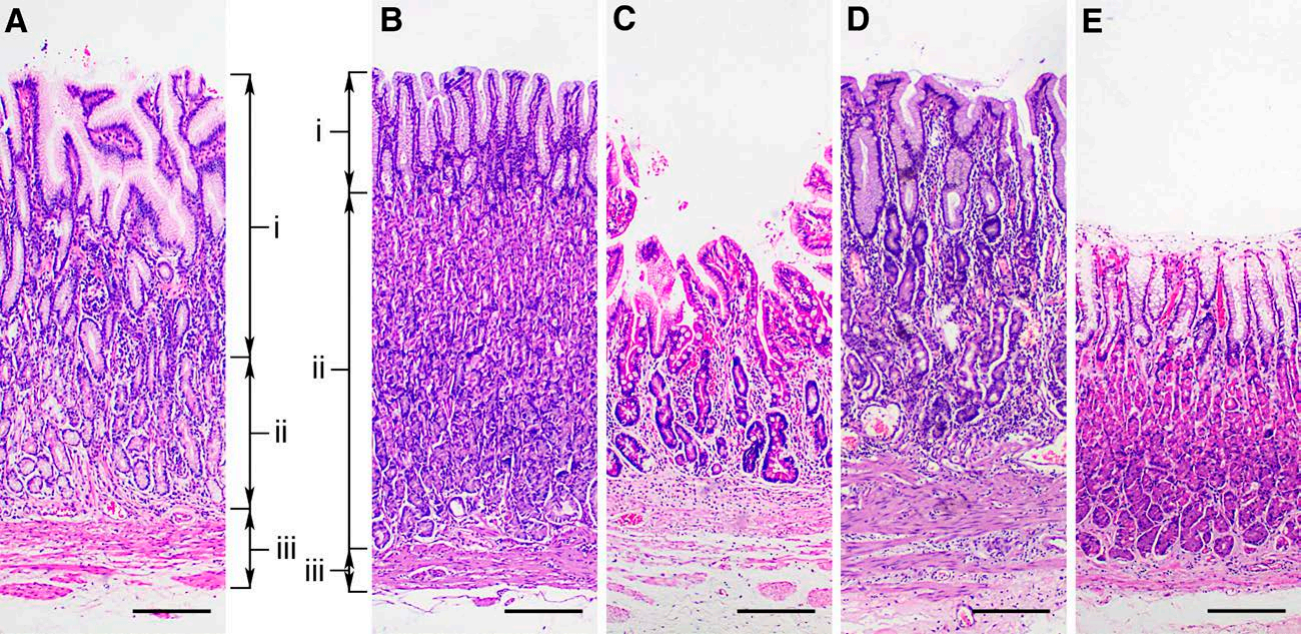
Morphometric measurements from gastric antral (A) and corpus mucosa (B–E). Foveolar length (i) was measured from the upper border of the superficial epithelium to the base of the gastric pit. Glandular length (ii) was defined as the distance between the bottom of the foveolae and upper border of musculus mucosae. Musculus mucosae thickness (iii) was measured from the upper edge to the lower edge of the musculus mucosae. Total mucosal thickness was the sum of the above 3 values. Compared with non-atrophic corpus mucosa (B), foveolar length and musculus mucosae thickness were increased and the glandular length was decreased in atrophic corpus mucosa (C and D), while the total mucosal thickness was variable or no change (C and D). E showed total mucosal thickness thinning without histopathological atrophy (Hematoxylin and eosin staining, 40× magnification, bar = 200 μm).

Considering the patchy distribution of atrophic mucosa in patients with atrophic gastritis,^[1,8,16]^ the thinnest area of the total mucosal thickness on a section was selected as the region of interest and demarcated with a marker.^[20]^ The region should have an intact epithelium and avoid the areas as follows: gastric sulci, lymphoid follicles, dysplasia and neoplasm. In the region of interest of each section, 3 length parameters should be measured (Fig. 1A and B): foveolar length, which was measured from the upper border of the superficial epithelium to the base of the gastric pit,^[18]^ glandular length, which was defined as the distance between the bottom of the foveola and upper border of musculus mucosae, and musculus mucosae thickness, which was defined as the distance from the upper edge to the lower edge of the musculus mucosae. Total mucosal thickness was the sum of the above 3 values. Foveolar/foveolar and glandular length ratio was calculated. The measurements were performed by 2 pathologists (X.-M.L. and Q.W.) on sections vertical to the mucosal surface using an image analysis system (ToupView; Hangzhou Touptek Photonics Co. Ltd., Hangzhou, China). The sections should be ignored if the orientation is not vertical. The average value was considered as the representative value.

### 2.4. Pathological assessment

For accurate assessment of mucosal thickness, all tissues were cut vertically into 5-mm slices, embedded in paraffin, sliced into 5-μm sections and stained with hematoxylin and eosin. In the region of interest of each section, atrophy was scored as a percentage of atrophic glands (no atrophy = 0%, score = 0; mild atrophy = 1–30%, score = 1; moderate atrophy = 31–60%, score = 2; and severe atrophy > 60%, score = 3).^[21]^ Pseudo-pyloric metaplasia and intestinal metaplasia are classified as metaplasia. Nonmetaplastic and metaplastic atrophy were considered together.^[21]^ All the specimens were scored by 2 experienced pathologists (X.-M.L. and Q.W.) for degrees of inflammation, activity, metaplasia, and *H pylori* density according to the visual analogue scale of the updated Sydney system (0 = none or minimal; 1 = mild; 2 = moderate; 3 = severe).^[4]^ The 2 pathologists, blinded to any clinical and endoscopic information, jointly examined all the specimens and reached a consensus on the score of each section. All pathological evaluations were performed in the mucosa at the region of interest.

### 2.5. Statistical analysis

The data were summarized using descriptive statistics, reporting the mean and standard deviation of normal distribution or median and interquartile ranges of skewed distribution for continuous variables, and proportions for categorical variables. Multiple comparison was performed with Kruskal–Wallis test and Mann–Whitney *U* test with Bonferroni corrections. Spearman′s correlation coefficient (*r*_s_) was calculated to evaluate the correlation. Regarding the diagnostic performance for mucosal atrophy, receiver operating characteristics (ROC) curve analysis was implemented and the area under the ROC curve (AUC), sensitivities, specificities, and cutoff values were calculated. The Youden index determined the optimal cutoff value. All analyses were performed using SPSS software (version 22.0; SPSS Inc., Chicago, IL). A 2-tailed *P* value < .05 was considered statistically significant.

## 3. Results

### 3.1. Baseline characteristics

A total of 401 surgical patients were enrolled in the study. The median age was 68.0 years (interquartile range, 59.0, 73.0; range 22–85 years), and 72.6% were male. 1098 corpus specimens and 405 antral specimens were obtained. 85.9% (943/1098) corpus sections and 83.0% (336/405) antral sections were technically feasible for morphometric and pathological assessment, after eliminating the ineligible sections such as epidermal exfoliation, tissue dissolution, atypical hyperplasia, and tumor. The overall eligibility rate of the sections was 85.1% (1279/1503).

### 3.2. Morphometric results and histological findings of corpus and antral mucosa

The morphometric results and histological findings of corpus mucosa were showed in Table 1 and Figure 1B–E. Among 943 corpus specimens, 71.3% (672/943) sections showed no atrophy (grade 0), and 8.9% (84/943), 9.3% (88/943) and 10.5% (99/943) sections showed atrophy grades 1, 2, and 3, respectively. Spearman correlation analysis showed that foveolar length and musculus mucosae thickness were weakly positively correlated with the atrophy degree (*P* < .05). Conversely, glandular length and total mucosal thickness were weakly negatively correlated with the atrophy degree (*P* < .05). The correlation coefficients were shown in Table 1. Multiple comparison suggested that the foveolar length and musculus mucosae thickness increased in atrophic subgroups (grade 2 or 3) compared with non-atrophic subgroups (grade 0). On the contrary, the glandular length (grades 1, 2 or 3 subgroups) and total mucosal thickness (grade 3 subgroup) decreased in the atrophic mucosa (Fig. S1A–H, Supplemental Digital Content, http://links.lww.com/MD/I764).

**Table 1:** Morphometric results and histological findings of corpus mucosa

Variables	Atrophy grade (n = 943)				Spearman correlation		
	0 (n = 672)	1 (n = 84)	2 (n = 88)	3 (n = 99)	*r*_s_	95% CI	*P* value
Foveolar length (mm)	0.29 (0.21, 0.39)	0.32 (0.24, 0.40)	0.38 (0.28, 0.47)	0.39 (0.30, 0.53)	0.231	0.168 to 0.292	<.001
Glandular length (mm)	0.55 (0.44, 0.68)	0.47 (0.35, 0.61)	0.38 (0.27, 0.49)	0.33 (0.26, 0.46)	−0.399	−0.453 to −0.342	<.001
Musculus mucosae thickness (mm)	0.08 (0.05, 0.12)	0.09 (0.05, 0.13)	0.10 (0.08, 0.16)	0.12 (0.08, 0.19)	0.224	0.161 to 0.286	<.001
Total mucosal thickness (mm)	0.95 (0.80, 1.12)	0.91 (0.76, 1.04)	0.92 (0.72, 1.09)	0.86 (0.70, 1.05)	−0.114	−0.178 to −0.048	<.001
Foveolar/foveolar and glandular length ratio	0.35 (0.28, 0.43)	0.40 (0.33, 0.50)	0.50 (0.39, 0.57)	0.54 (0.45, 0.62)	0.438	0.384 to 0.490	<.001
Inflammation grade	1 (1, 1)	2 (1, 2)	3 (2, 3)	3 (2, 3)	0.665	0.627 to 0.700	<.001
Activity grade	0 (0, 0)	0 (0, 1)	0 (0, 1)	0 (0, 1)	0.269	0.207 to 0.329	<.001
Metaplasia grade	0 (0, 0)	0 (0, 0)	0 (0, 0)	3 (0, 3)	0.600	0.556 to 0.641	<.001
*Helicobacter pylori* density	0 (0, 0)	0 (0, 0)	0 (0, 1)	0 (0, 0)	0.121	0.056 to 0.186	<.001

Data expressed as median and interquartile range (IQR). Four grades: 0 = none or minimal, 1 = mild, 2 = moderate, and 3 = severe.

The correlation coefficient (*r*_s_) between the mucosal thickness or histological scores and atrophy degrees was calculated by Spearman rank correlation.

CI = confidence interval.

The morphometric results and histological findings of antral mucosa were provided in Table 2. Among 336 antral specimens, 26.8% (90/336) sections showed no atrophy (grade 0), and 9.5% (32/336), 33.3% (112/336) and 30.4% (102/336) sections showed atrophy grade 1, 2 and 3, respectively. The foveolar length was very weakly negatively correlated with the atrophy degree (*r*_s_ = −0.147, *P* < .05). No correlation was observed between glandular length, musculus mucosae thickness or total mucosal thickness and the atrophy degree (*P* > .05). The correlation coefficients were exhibited in Table 2. Multiple comparison revealed that the foveolar length in atrophic subgroups (grade 3) decreased compared with non-atrophic subgroups (grade 0). There were no statistical differences between non-atrophic (grade 0) and atrophic subgroups (grades 1, 2 or 3) in glandular length, musculus mucosae thickness and total mucosal thickness (Fig. S2A–H, Supplemental Digital Content, http://links.lww.com/MD/I765).

**Table 2:** Morphometric results and histological findings of gastric antral mucosa.

Variables	Atrophy grade (n = 336)				Spearman correlation		*P* value
	0 (n = 90)	1 (n = 32)	2 (n = 112)	3 (n = 102)	*r*_s_	95% CI	
Foveolar length (mm)	0.51 (0.38, 0.69)	0.43 (0.31, 0.54)	0.45 (0.36, 0.59)	0.43 (0.35, 0.52)	−0.147	−0.253 to −0.038	.007
Glandular length (mm)	0.39 (0.26, 0.48)	0.35 (0.23, 0.48)	0.34 (0.25, 0.47)	0.37 (0.28, 0.46)	−0.009	−0.119 to 0.101	.867
Musculus mucosae thickness (mm)	0.14 (0.11, 0.22)	0.13 (0.11, 0.20)	0.14 (0.09, 0.23)	0.18 (0.12, 0.23)	0.067	−0.043 to 0.176	.219
Total mucosal thickness (mm)	1.09 (0.85, 1.32)	0.95 (0.83, 1.13)	1.00 (0.81, 1.20)	0.99 (0.83, 1.22)	−0.088	−0.196 to 0.022	.107
Foveolar/foveolar and glandular length ratio	0.59 (0.51, 0.67)	0.55 (0.51, 0.66)	0.56 (0.47, 0.66)	0.54 (0.48, 0.62)	−0.114	−0.221 to −0.004	.037
Inflammation grade	2 (1, 3)	3 (2, 3)	3 (2, 3)	3 (2, 3)	0.266	0.161 to 0.366	<.001
Activity grade	0 (0, 0)	0 (0, 0)	0 (0, 1)	0 (0, 1)	0.147	0.038 to 0.253	.007
Metaplasia grade	0 (0, 0)	0 (0, 0)	0 (0, 2)	3 (2, 3)	0.741	0.687 to 0.787	<.001
*Helicobacter pylori* density	0 (0, 0)	0 (0, 0)	0 (0, 0)	0 (0, 0)	−0.043	−0.152 to 0.068	.433

Data were expressed as median and interquartile range (IQR). Four grades: 0 = none or minimal, 1 = mild, 2 = moderate, and 3 = severe.

The correlation coefficient (*r*_s_) between the mucosal thickness or histological scores and atrophy degrees was calculated by Spearman rank correlation.

CI = confidence interval.

### 3.3. Diagnostic performance of total mucosal thickness for different atrophy degree

**Figure 2.**
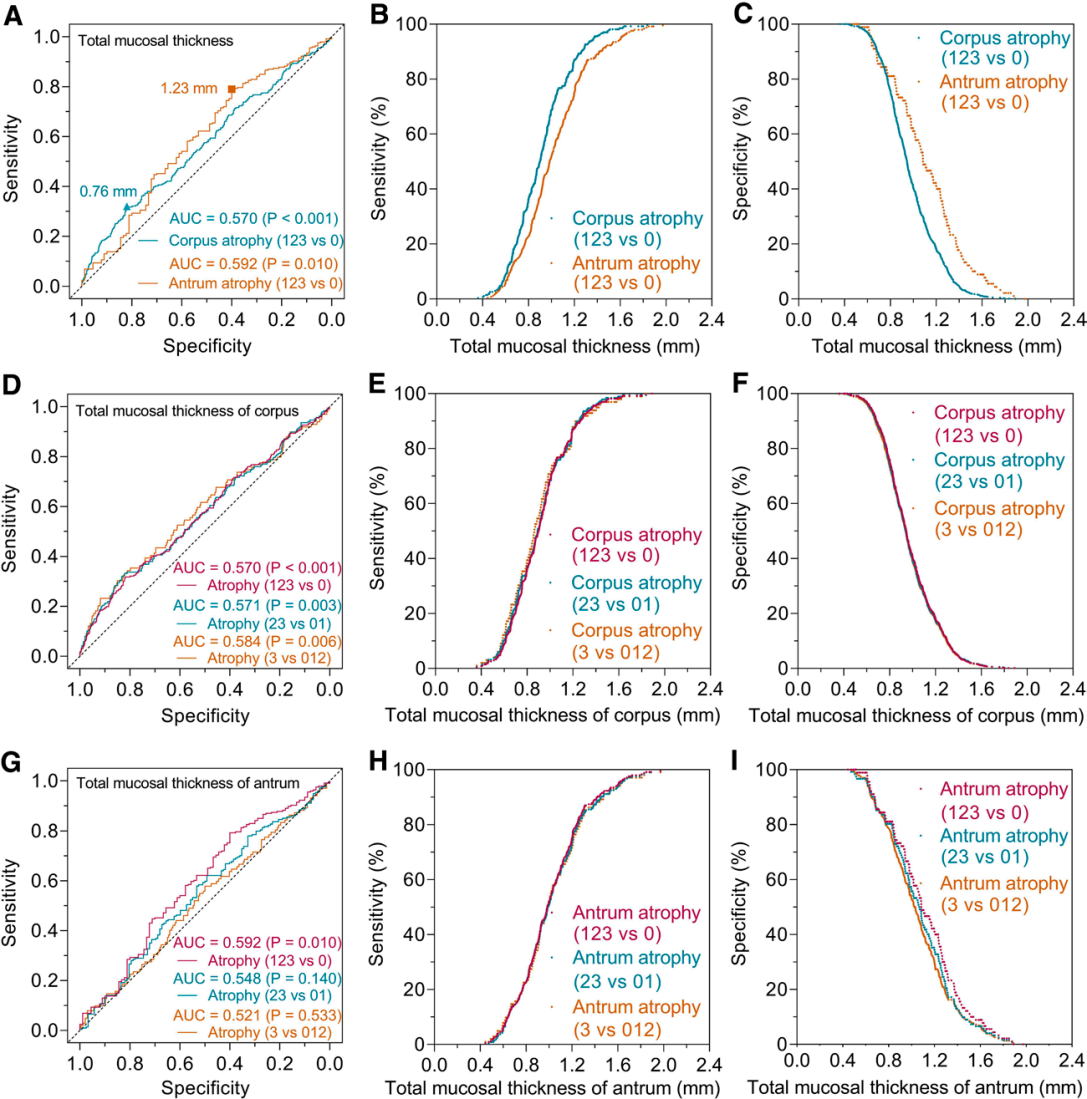
ROC curves for diagnosing various atrophy by total mucosal thickness in gastric corpus and antrum. For discriminating between atrophic (grade 1 + 2+3) and non-atrophic (grade 0) mucosa (A), the optimal cutoff value in gastric corpus was 0.76 mm with a sensitivity of 32%, specificity of 82%, and AUC was 0.570 (*P* < .001). The corresponding value in gastric antrum was 1.23 mm with a sensitivity of 79%, specificity of 40%, and AUC was 0.592 (*P* = .010). In the corpus mucosa (D), ROC analysis showed that the AUCs were 0.570 (*P* < .001), 0.571 (*P* = .003), and 0.584 (*P* = .006) for the atrophy (grade 1 + 2+3 vs 0), moderate and severe (grade 2 + 3 vs 0 + 1), and severe (grade 3 vs 0 + 1+2) atrophy, respectively. In the antral mucosa (G), ROC analysis showed that the AUCs were 0.592 (*P* = .010), 0.548 (*P* = .140), and 0.521 (*P* = .533) for the atrophy (grade 1 + 2+3 vs 0), moderate and severe (grade 2 + 3 vs 0 + 1), and severe (grade 3 vs 0 + 1+2) atrophy, respectively. B, C, E, F, H and I showed the trends and differences in the sensitivity and specificity with total mucosal thickness change. AUC = area under the curve, ROC = receiver operating characteristic.

The pathologic results were taken as the “gold standard” of mucosal atrophy. Atrophic mucosa was subdivided into mild, moderate and severe atrophy subgroups. ROC analysis (Fig. 2A and D, Table S1, Supplemental Digital Content, http://links.lww.com/MD/I766) showed that the corpus total mucosal thickness with less than 0.76 mm for assessing corpus atrophy (grade 1 + 2+3 vs 0) had a performance of AUC = 0.570 (95% confidence interval [CI], 0.529–0.611, *P* < .001), sensitivity = 32%, specificity = 82%. The corresponding values were AUC = 0.571 (95% CI, 0.523–0.619, *P* = .003), sensitivity = 33%, specificity = 82% for moderate and severe corpus atrophy (grade 2 + 3 vs 0 + 1). To predict severe corpus atrophy (grade 3 vs 0 + 1+2), the optimal cutoff value was 0.75 mm, and the corresponding values were AUC = 0.584 (95% CI, 0.520–0.648, *P* = .006), sensitivity = 33%, specificity = 82%. The scatter diagram showed that the sensitivity values of different corpus atrophy degrees were nearly identical (Fig. 2B and E), and the specificity values were also almost identical (Fig. 2C and F).

The antral total mucosal thickness with less than 1.23 mm for assessing antral atrophy (grade 1 + 2+3 vs 0) had a performance of AUC = 0.592 (95% CI, 0.521–0.662, *P* = .010), sensitivity = 79%, specificity = 40% (Fig. 2A and G, Table S2, Supplemental Digital Content, http://links.lww.com/MD/I767). The AUC values were 0.548 (95% CI, 0.484–0.613, *P* = .140) for moderate and severe atrophy, and 0.521 (95% CI, 0.454–0.589, *P* = .533) for severe atrophy, suggesting antrum total mucosal thickness was not statistically significant for moderate to severe atrophy. The scatter diagram showed the trends and differences in the sensitivity and specificity with total mucosal thickness change (Fig. 2H and I).

### 3.4. Comparison of mucosal thickness after activity stratification

The activity of gastric mucosa may cause changes in mucosal thickness and affect the diagnostic efficacy. Thus, all sections were further stratified into active or inactive groups according to pathologic assessments. The differences of corpus mucosal thickness between active and inactive groups are summarized in Table S3, Supplemental Digital Content, http://links.lww.com/MD/I768. For each atrophy degree, the corpus mucosa in majority of the active groups was thicker than that in the inactive groups, and partial differences reached statistically significance. For instance, in the non-atrophic corpus mucosa, the foveolar length and total mucosal thickness in active groups were longer than that in inactive groups (*P* < .05). In moderate atrophic corpus mucosa, the statistical differences were also observed in glandular length, musculus mucosae thickness and total mucosal thickness. However, in the antral mucosa (Table S4, Supplemental Digital Content, http://links.lww.com/MD/I769), there were no statistical differences between active and inactive groups in all mucosal thickness parameters.

### 3.5. Correlation between mucosal thickness and atrophy degree after activity stratification

Morphometric results of the corpus mucosa are depicted in Table S3, Supplemental Digital Content, http://links.lww.com/MD/I768. After activity stratification, there was no correlation between foveolar length and atrophy degrees in the activity group (*P* = .158), and the correlation trends in other length parameters were consistent with those before activity stratification. The correlation coefficients were exhibited in Table S3, Supplemental Digital Content, http://links.lww.com/MD/I768.

Morphometric results of the antral mucosa were demonstrated in Table S4, Supplemental Digital Content, http://links.lww.com/MD/I769. The correlation between the mucosal thickness parameters and the atrophy degrees was consistent before and after activity stratification. The correlation coefficients were presented in Table S4, Supplemental Digital Content, http://links.lww.com/MD/I769.

### 3.6. Effect of mucosal activity on diagnostic performance for atrophy

**Figure 3.**
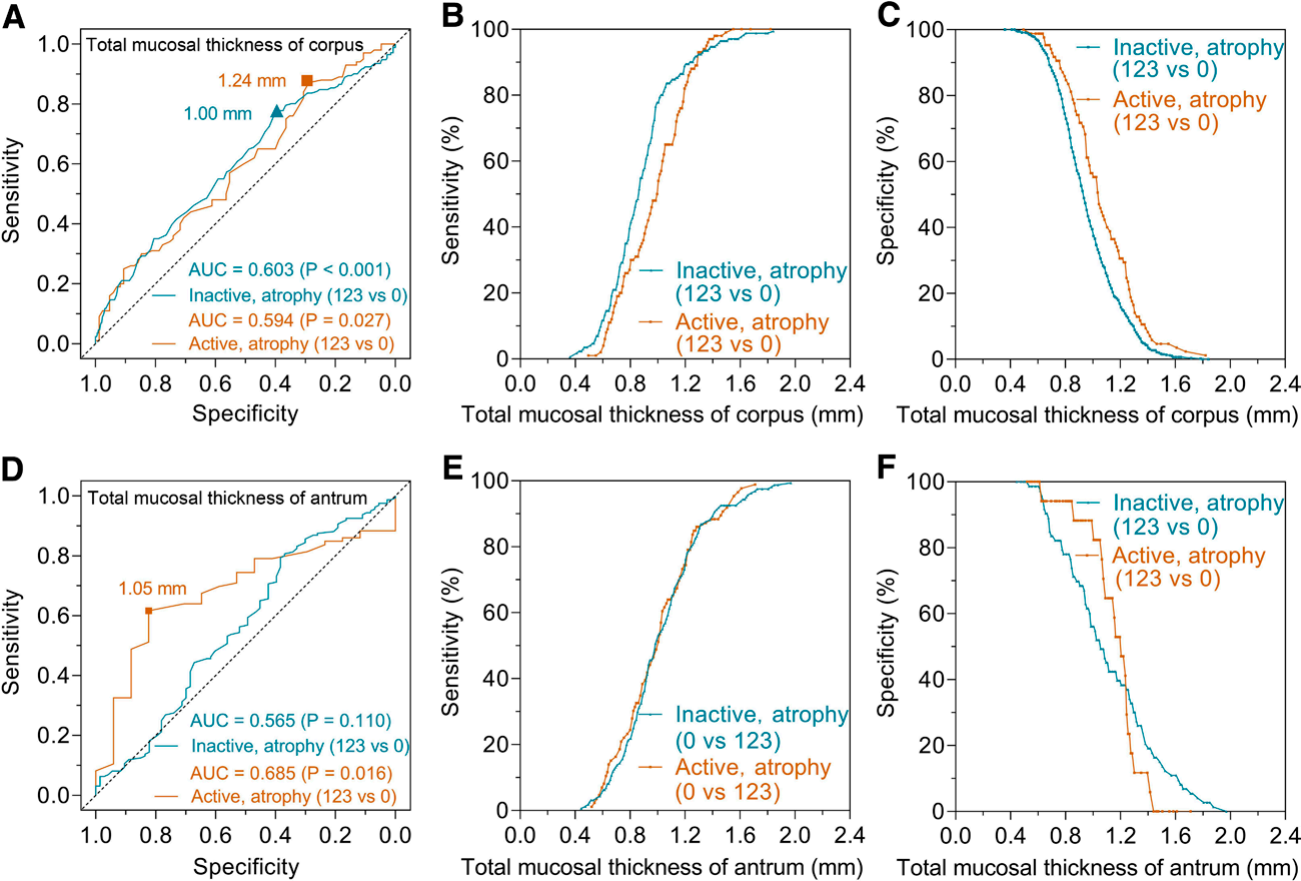
ROC curves for diagnosing atrophy by total mucosal thickness in active and inactive gastric mucosa. In the corpus mucosa (A), ROC analysis showed that the AUCs for atrophic mucosa (grade 1 + 2+3 vs 0) were 0.594 (*P* = .027) and 0.603 (*P* < .001) in active and inactive groups, respectively. In the antral mucosa (D), ROC analysis showed that the AUCs were 0.685 (*P* = .016) and 0.565 (*P* = .110) in active and inactive groups, respectively. B, C, E and F showed the trends and differences in the sensitivity and specificity with total mucosal thickness change. AUC = area under the curve, ROC = receiver operating characteristic.

In the active corpus mucosa (Fig. 3A), the AUC of total mucosal thickness for predicting corpus atrophy (grade 1 + 2+3 vs 0) was 0.594 (95% CI, 0.513–0.668, *P* = .027). The corresponding value was 0.603 (95% CI, 0.553–0.652, *P* < .001) in the inactive corpus mucosa. There was no statistical difference in AUCs between the active and inactive corpus mucosa (0.594 vs 0.603, *P* = .854). The scatter diagram showed the trends and differences in the sensitivity and specificity with total mucosal thickness change (Fig. 3B and C).

In the active antral mucosa (Fig. 3D), the AUC of total mucosal thickness for predicting antral atrophy (grade 1 + 2+3 vs 0) was 0.685 (95% CI, 0.564–0.806, *P* = .016). The corresponding value was 0.565 (95% CI, 0.483–0.648, *P* = .110) in the inactive antral mucosa. The scatter diagram showed the trends and differences in the sensitivity and specificity with total mucosal thickness change (Fig. 3E and F).

## 4. Discussion

In the present study, surgical stomach specimens were collected to investigate the morphometric features of the full-thickness gastric mucosa. The results showed that the foveolar length and musculus mucosae thickness in the corpus mucosa increased as atrophy degree aggravated, conversely, the glandular length and total mucosal thickness decreased. In the antral mucosa, the foveolar length was very weakly negatively correlated with the atrophy degree, whereas, the glandular length, musculus mucosae thickness and total mucosal thickness did not correlate with the increasing atrophy degree.

Adequate gastric mucosa tissues are crucial to approach a reliable morphological assessment. At present, endoscopic biopsy is the primary method to obtain the samples, while it has the weakness of insufficient biopsy depth. Among the specimens obtained by gastric biopsy, Kim et al^[17]^ found that 36.0% (140/389) corpus specimens and 50.4% (196/389) antral specimens had no intact foveolar and glandular layers for pathological evaluation. In the Saghier et al^[18]^ study, more than 200 gastric biopsies were screened, and only 93 (<42.3%) slides had adequate foveolar and glandular layers to meet the requirements of morphological measurement. These results indicated that the remaining eligible sections were not enough to represent the real morphological features of gastric mucosa. In addition, previous studies also failed to evaluate the changes of musculus mucosae. Moreover, crush artifacts in gastric biopsy specimens could decrease the quality of specimens,^[22]^ and incorrect orientation of the biopsy samples inevitably affected the morphometric evaluation.^[1,23]^ Therefore, to avoid the above difficulties of biopsy tissues, surgical gastric specimens with full-thickness mucosa were utilized in the study.

Gastric atrophy is defined as the loss of “appropriate glands.”^[3,4]^ Atrophic gastritis is a chronic process, in which the glands may be replaced with connective tissue or a metaplastic epithelium. It is generally accepted that the mucosal thickness of atrophic gastritis becomes thin.^[1,4]^ Based on the corpus morphometric results (Table 1), we confirmed that the full-thickness gastric mucosa did become thinner with the atrophic aggravation, yet the correlation coefficient was very low (*r*_s_ = −0.114, *P* < .001). Besides, we also revealed that the decrease in corpus full-thickness mucosa was solely caused by the thinned glandular layer. The thinning tendency of corpus full-thickness mucosa would be attenuated by the gradual increase of foveolar length and musculus mucosae thickness. By contrast, our results did not show a decrease in antral total mucosal thickness with increasing atrophy degree. Similarly, the antral glandular layer did not become thinner with increasing atrophy, which was consistent with the results reported previously by Ruiz et al^[19]^ In a word, the change trends of corpus mucosa with atrophy differed from antral mucosa.

Moreover, the effect of mucosal activity on corpus and antral mucosal thickness was also different. Morphometric results demonstrated that activity elimination could decrease the total mucosal thickness in atrophic and non-atrophic corpus mucosa, which was consistent with prior studies^[24,25]^ and agreed with the endoscopic mucosal appearance after *H pylori* eradication. On the contrary, no statistical differences were found in all antral morphometric parameters between the active and inactive mucosa (Table S4, Supplemental Digital Content, http://links.lww.com/MD/I769), which suggested that mucosal activity regression could not affect the thickness of gastric antral mucosa.

Submucosal vascular pattern is one of the well-known endoscopic signs to discern mucosal atrophy under gastroscopy. The thinning of gastric mucosal thickness to a cutoff value will cause this sign to appear. In the in vivo study of endoscopic atrophic signs,^[26]^ ROC analysis found that the AUCs of vascular pattern for identifying corpus and antral atrophy were 0.77 and 0.62, respectively, which were higher than our results (0.57 for corpus and 0.59 for antrum, respectively). In their study, they have pointed out that the biopsy sites did not exactly correspond with the endoscopic sign points. Also, the different amount of air insufflation during gastroscopy would affect the gastric mucosal transparency and even cause normal vascular ramifications visible,^[7]^ confusing the judgment of real endoscopic atrophy signs. Furthermore, the different gastric wall may vary in the ability to stretch when air inflated.^[27]^ Therefore, we used surgical gastric specimens for morphometric evaluation in vitro. Gastric mucosal thickness and atrophy degree were compared in the area of interest of the sections, providing objective evidence for the diagnostic efficacy. The results confirmed that the full-thickness gastric mucosa was not a good classifier for both corpus and antral mucosal atrophy. We also calculated the diagnostic performance of total mucosal thickness for the different degrees of corpus atrophy, and all the AUCs were less than 0.6 (Fig. 2), indicating that this indicator was not a practical parameter for predicting different atrophy. The previous study by Nomura et al^[26]^ also showed that no single endoscopic sign, including submucosal vascular pattern, could precisely predict atrophy, and it was necessary to combine multiple endoscopic findings to improve the diagnostic accuracy.

The resolution of mucosal activity can shorten the total mucosal thickness of gastric corpus, which may affect the diagnostic efficacy for atrophy. As the scatterplots (Fig. 3B and C) showed that, after the mucosal activity disappearance, the sensitivity in diagnosing corpus atrophy would increase, while the specificity was decreased. As a result, the AUC of the inactive group was only slightly higher than that of the active group (Fig. 3A), and the difference did not reach a statistical difference (0.603 vs 0.594, *P* = .854), suggesting that the disappearance of mucosal activity could not improve the diagnostic efficacy.

Previous studies have investigated the sensitivity of submucosal vessels visibility in the diagnosis of corpus and antrum atrophy.^[15,26,28]^ Theoretically, the penetration depth of light illumination on corpus and antral mucosa is almost consistent.^[29]^ So, the different thickness of corpus and antral mucosa would produce distinct sensitivity. Based on our ROC results, the scatter diagram (Fig. 2B) showed that the sensitivity of the corpus was higher than that of the antrum, which implied that submucosal vessels visibility could more readily discern the corpus atrophy. Indeed, Redéen et al^[28]^ found that the sensitivity of the visible vessels for predicting moderate to severe atrophy was 48% in the corpus and 14% in the antrum. Nomura et al^[26]^ also found that the sensitivity in the corpus (79%) was higher than that in the antrum (61%). In contrast to the study by Eshmuratov et al,^[15]^ the sensitivity of the antrum (61.5%) was higher than that of the corpus (46.8%), which was inconsistent with our results. The reasons were complicated for the divergent sensitivity. Besides the different amount of air insufflation, the endoscopists′ subjectivity in discerning atrophy signs might be another inevitable factor. To identify submucosal blood vessels, the total mucosal thickness must be within the penetration depth of light illumination.^[29]^ In view of the in vitro nature of our current study, the optimal cutoff values of total mucosal thickness for discerning submucosal blood vessels had not been obtained and the corresponding sensitivity cannot be achieved.

Previous studies^[28]^ have revealed that the sensitivity and specificity of corpus vascular visibility for judging moderate to severe atrophy were 48% and 87%, and the corresponding values for severe atrophy were 80% and 87%, respectively. But, based on our morphometric results (Fig. 2E and F), the specificity and sensitivity of the same mucosal thickness for predicting the different degrees of corpus atrophy should be nearly identical. Indeed, more severe corpus atrophy led to thinner glandular thickness. However, gradually increasing the foveolar length and musculus mucosae thickness would reduce the thinning trend with atrophy. The total mucosal thickness showed only a very weak negative correlation with atrophy degrees (*r*_s_ = −0.114, Table 1). It was possible that the slight change in total mucosal thickness with atrophy degrees was not yet sufficient to cause significant differences in sensitivity and specificity. Similarly, the endoscopic signs did not correspond to the pathological loci in this study.^[28]^ We also realized that the differences between in vivo endoscopic observations and in vitro metrological findings were not negligible. Therefore, it is necessary to obtain full-thickness gastric mucosa with jumbo forceps,^[30]^ and then point-to-point endoscopic atrophy sign and morphometric analysis is further required.

There were several limitations to our study. First, our conclusions were drawn from gastric cancer patients and should be interpreted with caution to non-gastric cancer populations. Second, gastric mucosal inflammation might affect the grading of atrophy degree, especially in antral mucosa. We stratified the samples by mucosal activity and atrophy degrees to reduce the effect. Last, *H pylori*-naïve normal gastric mucosa was not included in the study.

## 5. Conclusions

Overall, the total mucosal thickness of the gastric body and antrum showed different trends with the degree of atrophy. The thinning tendency in corpus total mucosal thickness can be attenuated by the gradual increase of foveolar length and musculus mucosae thickness. On the contrast, the trend did not be revealed in the antral mucosa. Furthermore, morphological study showed that full-thickness mucosal thickness was not a practical indicator for predicting mucosal atrophy either in the corpus or antrum. Therefore, it is necessary to combine multiple endoscopic atrophy signs to improve the diagnostic accuracy of atrophy in clinical practice. Eventually, the diagnosis of atrophic gastritis should be confirmed by histopathology.

## Author contributions

**Data curation:** Xue-Mei Lin, Li Wang, Chun-Hui Xi, Jun Wang.

**Formal analysis:** Xue-Mei Lin, Li Wang, Chun-Hui Xi, Cong Yuan.

**Funding acquisition:** Cong Yuan.

**Investigation:** Xian-Fei Wang, Qiong Wang.

**Methodology:** Xue-Mei Lin, Qiong Wang.

**Project administration:** Cong Yuan.

**Resources:** Xue-Mei Lin, Li Wang, Xian-Fei Wang.

**Software:** Cong Yuan.

**Validation:** Cong Yuan.

**Visualization:** Xue-Mei Lin, Li Wang, Chun-Hui Xi, Jun Wang.

**Writing – original draft:** Xue-Mei Lin.

**Writing – review & editing:** Xian-Fei Wang, Cong Yuan.

## Supplementary Material
