# Supplementary material for: Morphometric features of gastric mucosa in atrophic gastritis: A different pattern between corpus and antrum
Source: Medicine (Baltimore). 2022 Apr 7;102(14):e33480. doi: 10.1097/MD.0000000000033480 (PMC10082242; doi:10.1097/MD.0000000000033480)

Supplementary Fig. 1. Comparison of morphometric results and histological findings of corpus mucosa divided by atrophy degree. Box plots denoted the medians (black dot) and the interquartile ranges (IQRs). The whiskers of each boxplot were the lowest or highest datum still within 1.5 IQR of the lower or upper quartile. Violin plot width was based on a Gaussian kernel density estimate of the data. There were statistical differences between the non-atrophic (grade 0) and one or more atrophic subgroups (grade 1, 2 or 3) in foveolar length (A), glandular length (B), musculus mucosae thickness (C), and total mucosal thickness (D). Similar statistical differences were also found in inflammation (E), activity (F), metaplasia (G), and *H. pylori* density (H). *$P < .05$ by Kruskal-Wallis test.

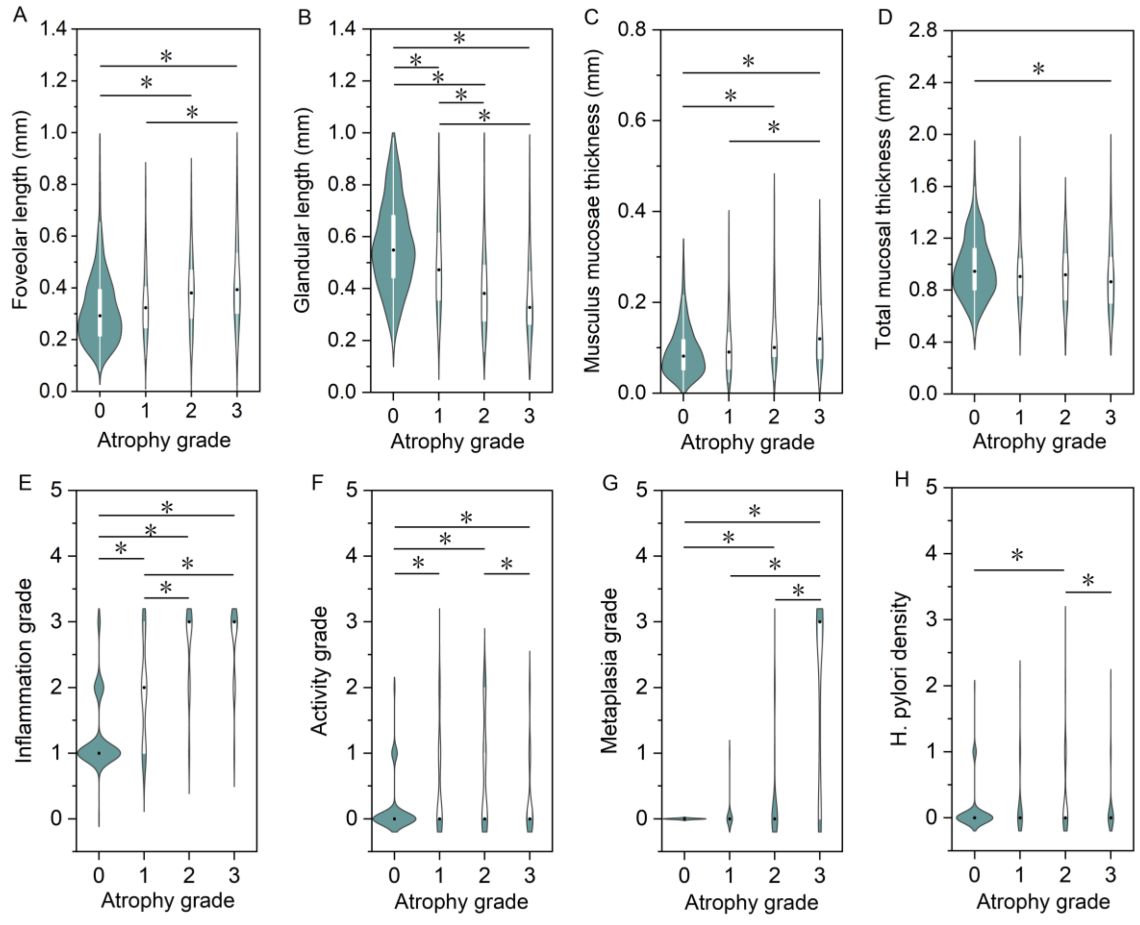

Supplement: Supplementary file 1 [file medi-102-e33480-s001.pdf]
