# Supplementary material for: Morphometric features of gastric mucosa in atrophic gastritis: A different pattern between corpus and antrum
Source: Medicine (Baltimore). 2022 Apr 7;102(14):e33480. doi: 10.1097/MD.0000000000033480 (PMC10082242; doi:10.1097/MD.0000000000033480)

Supplementary Fig. 2. Comparison of morphometric results and histological findings of antral mucosa divided by atrophy degree. Violin and boxplots showed the medians, interquartile ranges and data distribution. There were statistical differences between the non-atrophic (grade 0) and one or more atrophic subgroups (grade 1, 2 or 3) in foveolar length (A), inflammation (E), activity (F) and metaplasia (G). No statistical differences between the non-atrophic and atrophic subgroups were discovered in glandular length (B), musculus mucosae thickness (C), and total mucosal thickness (D). **P* < .05 by Kruskal-Wallis test.

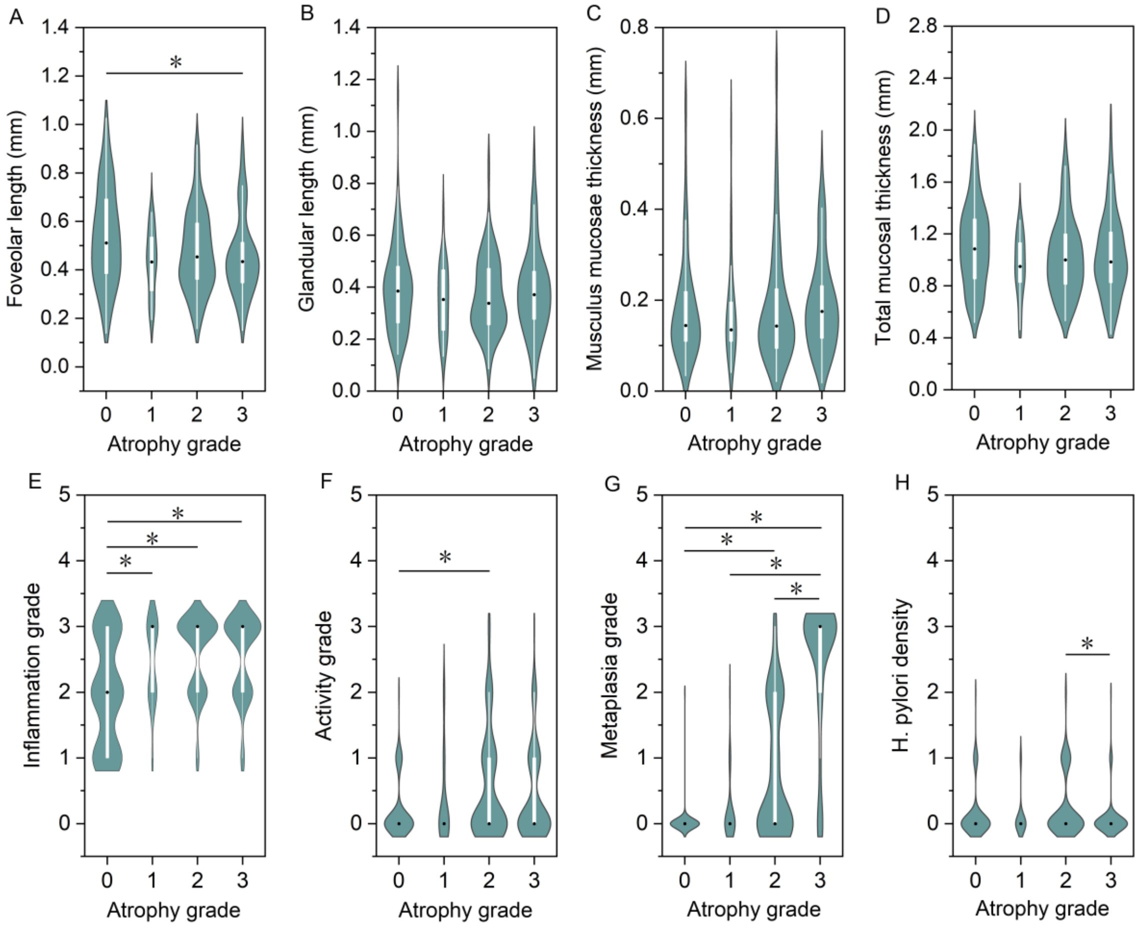

Supplement: Supplementary file 2 [file medi-102-e33480-s002.pdf]
