# Supplementary material for: Morphometric features of gastric mucosa in atrophic gastritis: A different pattern between corpus and antrum
Source: Medicine (Baltimore). 2022 Apr 7;102(14):e33480. doi: 10.1097/MD.0000000000033480 (PMC10082242; doi:10.1097/MD.0000000000033480)
Supplement: Supplementary file 3 [file medi-102-e33480-s003.pdf]

Supplementary Table 1 Receiver operating characteristic table of corpus total mucosal thickness in diagnosing different degrees of corpus atrophy

Cut-off value	Mild + moderate + severe vs absent‡			Moderate + severe vs absent + mild‡			Severe vs absent + mild + moderate‡		
(mm)	Sensitivity	Specificity	Youden index	Sensitivity	Specificity	Youden index	Sensitivity	Specificity	Youden index
0.70	0.21	0.89	0.10	0.22	0.88	0.10	0.25	0.87	0.12
**0.75†**	0.27	0.84	0.11	0.31	0.83	0.14	**0.33**	**0.82**	**0.15**
**0.76†**	**0.32**	**0.82**	**0.14**	**0.33**	**0.82**	**0.15**	0.35	0.8	0.15
0.80	0.36	0.76	0.12	0.37	0.75	0.12	0.39	0.74	0.13
0.90	0.5	0.58	0.08	0.51	0.57	0.08	0.55	0.57	0.12
0.95	0.59	0.49	0.08	0.59	0.48	0.07	0.64	0.48	0.12
1.00	0.69	0.40	0.09	0.68	0.39	0.07	0.71	0.39	0.10
1.10	0.77	0.28	0.05	0.77	0.27	0.04	0.78	0.27	0.05
1.20	0.87	0.18	0.05	0.87	0.18	0.05	0.88	0.17	0.05
1.23	0.89	0.16	0.05	0.89	0.16	0.05	0.90	0.16	0.06
1.25	0.89	0.15	0.04	0.90	0.14	0.04	0.90	0.14	0.04
1.30	0.93	0.10	0.03	0.94	0.1	0.04	0.92	0.10	0.02
1.40	0.96	0.05	0.01	0.96	0.05	0.01	0.94	0.04	-0.02

Sensitivity and specificity were calculated by receiver-operating characteristic analysis.

†The optimal cut-off values were determined by Youden index and shown in bold.

‡Gastric mucosal atrophy degree.

Vs, versus.
