# Supplementary material for: Morphometric features of gastric mucosa in atrophic gastritis: A different pattern between corpus and antrum
Source: Medicine (Baltimore). 2022 Apr 7;102(14):e33480. doi: 10.1097/MD.0000000000033480 (PMC10082242; doi:10.1097/MD.0000000000033480)
Supplement: Supplementary file 4 [file medi-102-e33480-s004.pdf]

Supplementary Table 2 Receiver operating characteristic table of antral total mucosal thickness in diagnosing different degrees of antral atrophy

Cut-off value	Mild + moderate + severe vs absent‡			Moderate + severe vs absent + mild‡			Severe vs absent + mild + moderate‡		
(mm)	Sensitivity	Specificity	Youden index	Sensitivity	Specificity	Youden index	Sensitivity	Specificity	Youden index
0.70	0.14	0.86	0.00	0.14	0.85	-0.01	0.16	0.86	0.02
0.75	0.18	0.84	0.02	0.18	0.84	0.02	0.19	0.83	0.02
0.76	0.19	0.84	0.03	0.19	0.84	0.03	0.19	0.82	0.01
0.80	0.23	0.81	0.04	0.23	0.80	0.03	0.23	0.79	0.02
0.90	0.37	0.73	0.10	0.36	0.70	0.06	0.34	0.66	0.00
0.95	0.45	0.68	0.13	0.44	0.62	0.06	0.44	0.59	0.03
1.00	0.52	0.61	0.13	0.51	0.56	0.07	0.52	0.53	0.05
**1.03†**	0.56	0.58	0.14	0.55	0.53	0.08	**0.58**	**0.50**	**0.08**
**1.08†**	0.62	0.53	0.15	**0.62**	**0.49**	**0.11**	0.61	0.44	0.05
1.10	0.64	0.49	0.13	0.63	0.43	0.06	0.62	0.40	0.02
1.20	0.75	0.41	0.16	0.74	0.34	0.08	0.72	0.29	0.01
**1.23†**	**0.79**	**0.40**	**0.19**	0.78	0.33	0.11	0.76	0.27	0.03
1.25	0.81	0.36	0.17	0.80	0.30	0.10	0.78	0.24	0.02
1.30	0.85	0.27	0.12	0.84	0.20	0.04	0.83	0.18	0.01
1.40	0.89	0.18	0.07	0.87	0.13	0.00	0.87	0.13	0.00

Sensitivity and specificity were calculated by receiver-operating characteristic analysis.

†The optimal cut-off values were determined by Youden index and shown in bold.

‡Gastric mucosal atrophy degree.

Vs, versus.
