# Supplementary material for: Morphometric features of gastric mucosa in atrophic gastritis: A different pattern between corpus and antrum
Source: Medicine (Baltimore). 2022 Apr 7;102(14):e33480. doi: 10.1097/MD.0000000000033480 (PMC10082242; doi:10.1097/MD.0000000000033480)
Supplement: Supplementary file 5 [file medi-102-e33480-s005.pdf]

Supplementary Table 3 Comparison of corpus mucosal thickness stratified by activity and atrophy grade

	Atrophic grade (*n* = active/inactive)				Spearman correlation		
Parameters (mm)	0 (*n* = 85/587)	1 (*n* = 32/52)	2 (*n* = 43/45)	3 (*n* = 25/74)	r_s	(CI 95%)	*P* value
Foveolar length							
Active (*n* = 185)	0.37 (0.26, 0.45)*	0.34 (0.24, 0.46)	0.40 (0.33, 0.51)	0.37 (0.29, 0.53)	0.104	-0.045 to 0.249	0.158
Inactive (*n* = 758)	0.28 (0.21, 0.38)	0.31 (0.24, 0.39)	0.34 (0.24, 0.45)	0.39 (0.30, 0.51)	0.216	0.145 to 0.285	< 0.001
Glandular length							
Active (*n* = 185)	0.58 (0.48, 0.73)	0.52 (0.36, 0.65)	0.43 (0.33, 0.50)*	0.34 (0.26, 0.49)	-0.453	-0.564 to -0.327	< 0.001
Inactive (*n* = 758)	0.55 (0.44, 0.68)	0.44 (0.35, 0.60)	0.34 (0.27, 0.46)	0.33 (0.26, 0.43)	-0.394	-0.454 to -0.330	0.000
Musculus mucosae thickness							
Active (*n* = 185)	0.08 (0.06, 0.13)	0.10 (0.07, 0.13)	0.12 (0.09, 0.18)*	0.12 (0.08, 0.23)	0.297	0.155 to 0.427	< 0.001
Inactive (*n* = 758)	0.08 (0.05, 0.12)	0.09 (0.05, 0.14)	0.09 (0.07, 0.15)	0.13 (0.08, 0.18)	0.179	0.107 to 0.250	< 0.001
Total mucosal thickness							
Active (*n* = 185)	1.04 (0.88, 1.25)*	1.00 (0.80, 1.13)	1.01 (0.75, 1.18)*	0.95 (0.73, 1.19)	-0.160	-0.301 to -0.012	0.029
Inactive (*n* = 758)	0.93 (0.79, 1.11)	0.87 (0.76, 0.98)	0.86 (0.72, 0.98)	0.86 (0.69, 1.01)	-0.149	-0.220 to -0.076	< 0.001

Data expressed as median and interquartile range (IQR). Four grades: 0 = none or minimal, 1 = mild, 2 = moderate, and 3 = severe.

Active group included activity grade 1, 2, and 3. Inactive group referred to activity grade 0.

The correlation coefficient (r_s) between the mucosal thickness and atrophy degrees was calculated by Spearman rank correlation.

*$p < 0.05$ active versus inactive groups at the same grade of atrophy by Mann-Whitney U test.

CI, confidence interval.
