# Supplementary material for: Morphometric features of gastric mucosa in atrophic gastritis: A different pattern between corpus and antrum
Source: Medicine (Baltimore). 2022 Apr 7;102(14):e33480. doi: 10.1097/MD.0000000000033480 (PMC10082242; doi:10.1097/MD.0000000000033480)
Supplement: Supplementary file 6 [file medi-102-e33480-s006.pdf]

Supplementary Table 4 Comparison of antral mucosal thickness stratified by activity and atrophy grade

	Atrophy grade (n = active/inactive)				Spearman correlation		
Parameters (mm)	0 (n = 17/73)	1 (n = 7/25)	2 (n = 44/68)	3 (n = 35/67)	r_s	(CI 95%)	*P* value
Foveolar length							
Active (n = 103)	0.57 (0.44, 0.71)	0.48 (0.43, 0.61)	0.41 (0.35, 0.60)	0.44 (0.35, 0.53)	-0.202	-0.386 to -0.003	0.040
Inactive (n = 233)	0.50 (0.36, 0.68)	0.42 (0.29, 0.53)	0.47 (0.36, 0.60)	0.42 (0.34, 0.51)	-0.129	-0.257 to 0.003	0.049
Glandular length							
Active (n = 103)	0.41 (0.36, 0.51)	0.40 (0.30, 0.48)	0.36 (0.24, 0.50)	0.35 (0.29, 0.46)	-0.163	-0.351 to 0.037	0.099
Inactive (n = 233)	0.38 (0.25, 0.47)	0.34 (0.23, 0.47)	0.33 (0.27, 0.47)	0.38 (0.27, 0.47)	0.043	-0.090 to 0.174	0.518
Musculus mucosae thickness							
Active (n = 103)	0.12 (0.09, 0.18)	0.12 (0.06, 0.15)	0.13 (0.08, 0.19)	0.17 (0.10, 0.27)	0.169	-0.031 to 0.356	0.088
Inactive (n = 233)	0.15 (0.11, 0.23)	0.16 (0.12, 0.21)	0.16 (0.11, 0.23)	0.18 (0.12, 0.23)	0.050	-0.083 to 0.181	0.448
Total mucosal thickness							
Active (n = 103)	1.2 (0.44, 0.71)	0.92 (0.84, 1.17)	1.01 (0.80, 1.21)	1.00 (0.77, 1.25)	-0.138	-0.329 to 0.062	0.163
Inactive (n = 233)	1.05 (0.84, 1.37)	0.97 (0.79, 1.12)	1.00 (0.82, 1.20)	0.98 (0.84, 1.21)	-0.064	-0.194 to 0.069	0.334

Data expressed as median and interquartile range (IQR). Four grades: 0 = none or minimal, 1 = mild, 2 = moderate, and 3 = severe.

Active group included activity grade 1, 2, and 3. Inactive group referred to activity grade 0.

The correlation coefficient (r_s) between the mucosal thickness and atrophy degrees was calculated by Spearman rank correlation.

No statistical differences were found between active and inactive groups at the same grade of atrophy by Mann-Whitney U test.

CI, confidence interval.
